# Methylation of PRDX3 Expression Alleviate Ferroptosis and Oxidative Stress in Patients with Osteoarthritis Cartilage Injury

**DOI:** 10.5152/ArchRheumatol.2025.11031

**Published:** 2025-05-28

**Authors:** Xia Zhao, Yixue Peng, Mengsong Wang, Qishuang Tan

**Affiliations:** 1 Institute of Respiratory and Co-occurring Diseases, West China Hospital of Sichuan University, Chengdu, China; 2 Anaesthesia Surgery Centre, West China Hospital of Sichuan University, Chengdu, China; 3 Department of Anesthesiology, West China Hospital of Sichuan University, Chengdu, China; 4 Research Base, West China Hospital of Sichuan University, Chengdu, China

**Keywords:** Cartilage injury, ferroptosis, METTL3, osteoarthritis, PRDX3

## Abstract

**Background/Aims:**

Osteoarthritis typically features cartilage degeneration, synovial fibrosis, and bone remodeling. While clinical Western medicine therapies can restore joint functions, long-term use may exacerbate cartilage damage. This study was designed to investigate the impact of peroxiredoxin 3 (PRDX3) on ferroptosis and oxidative stress in osteoarthritis cartilage injury and its potential mechanism.

**Materials and Methods:**

In the osteoarthritis model, the expression of PRDX3 was downregulated. Single-cell analysis revealed that the *PRDX3* gene was expressed in bone cells of osteoarthritis patients.

**Results:**

Sh-PRDX3 promoted osteoarthritis cartilage injury in the mouse model via the induction of oxidative stress. PRDX3 suppressed reactive oxygen species accumulation and mitochondria-dependent ferroptosis in the in vitro model or mice model of osteoarthritis. PRDX3 induced SIRT3 to reduce SIRT3 ubiquitin. Moreover, METTL3-mediated m6A modification decreases PRDX3 mRNA stability by YTHDF1 in the osteoarthritis cartilage injury model.

**Conclusion:**

These findings indicate that METTL3-mediated m6A modification decreases PRDX3 mRNA stability to relieve ferroptosis and oxidative stress in the model of osteoarthritis cartilage injury in a YTHDF1-dependent manner. Targeting METTL3 is thus a potentially effective therapeutic strategy for patients with osteoarthritis cartilage injury.

**Main Points**

- Sh-PRDX3 promoted osteoarthritis cartilage injury in the mouse model of osteoarthritis via the induction of oxidative stress.
- PRDX3 suppressed reactive oxygen species accumulation and mitochondria-dependent ferroptosis in model of osteoarthritis.
- PRDX3 induced SIRT3 to reduce SIRT3 ubiquitin in model of osteoarthritis.
- METTL3-mediated m6A modification decreases PRDX3 mRNA stability by YTHDF1 in the osteoarthritis cartilage injury model.

## Introduction

Osteoarthritis (OA) can lead to joint pain, swelling, and restricted joint movement in patients, which has a serious impact on the quality of life of middle-aged and elderly patients.^1,2^ Meanwhile, there is currently no drug capable of effectively halting or reversing the pathological process of OA.^3^ Consequently, it is highly significant to elucidate its pathogenesis and explore economic, safe, and effective early prevention and treatment approaches.^4^ In clinical settings, opioids, non-steroidal anti-inflammatory drugs, corticosteroids, and other medications are frequently utilized for the treatment of early to mid-stage osteoarthritis patients.^5^ Knee replacement surgery is employed to treat patients in the late stage of OA.^6^ Although medical and surgical treatments can ameliorate patient symptoms to a certain extent, neither can effectively repair the damaged cartilage of a patient.^7^

Knee osteoarthritis (KOA) comprises 70% to 80% of OA patients. In the context of an intensifying aging trend, the incidence of KOA has risen significantly, imposing a substantial burden on families and society. Consequently, the research and development of safe and effective drugs for KOA treatment has become an urgent problem that demands immediate resolution.^8-10^ The non-surgical treatments recommended by the International Osteoarthritis Research Society International (OARSI) are mainly oral non-steroidal anti-inflammatory drugs.^11^ Although these drugs can alleviate certain symptoms, long-term use may lead to numerous adverse reactions.^12^

Iron-dependent cell death, which is triggered by lipid peroxidation and reactive oxygen species (ROS) accumulation, represents a mode of cell death that plays a crucial role in diverse diseases, such as knee osteoarthritis (KOA).^13^ Research has shown that iron death inhibitors can upregulate the expression of matrix metalloproteinase-13 in chondrocytes, simultaneously downregulate the expression of collagen II in chondrocytes, and impede the progression of KOA.^14^ The level of ROS gradually elevates due to factors like age, inflammatory factors, and mechanical stimulation. Multiple inflammatory cytokines are implicated in the development of OA disease, causing chondrocytes to undergo senescence and apoptosis.^15^ Excessive ROS induces matrix-degrading proteases and cell-surface proteins; diminishes extracellular matrix synthesis, resulting in abnormal metabolism in cartilage and bone tissue; cartilage degeneration and degradation of cartilage; and aggravating the damage, apoptosis, and degeneration of chondrocytes and osteoblasts.^15^

Systemic iron overload can also lead to iron accumulation in guinea pig joints and lead to joint degeneration. Moreover, inhibiting ferroptosis can significantly ameliorate osteoarthritis, suggesting that the targeted regulation of chondrocyte ferroptosis and the accumulation of ferrous ions can initiate membrane lipid peroxidation reactions, and even evoke oxidative stress, promote the accumulation of malondialdehyde (MDA), and consequently cause cellular oxidative damage and death. Damage to the antioxidant system is regarded as a crucial factor contributing to the accumulation of lipid peroxidation.^16,17^ Liposome peroxidation can disrupt the composition and structure of cell membranes or modulate oxidative stress-related products such as ROS, MDA, and superoxide dismutase (SOD). These substances can react with DNA or proteins and further exert toxic effects.^18^

SIRT3, a deacetylase distributed in mitochondria, is related to various physiological and pathological processes within cells, such as aging, energy metabolism, cell apoptosis, stress, and inflammatory responses. It plays a role in maintaining mitochondrial function and is a crucial deacetylase for regulating protein acetylation in mitochondria. SIRT3 can prevent oxidative stress and protect cells from the impacts of aging and oxidative stress.

The Peroxiredoxin (PRDX) family is a novel and important protein family with antioxidant functions and is widely expressed in organisms. PRDX3 can promote the invasion of triple-negative breast cancer by up-regulating metalloproteinases, and hypoxia can also induce PRDX3 to enhance tumorigenesis and cell invasion of renal cell carcinoma.^19^ Besides peroxidase activity, PRDX6 also shows unique phospholipase A2 (PLA2) activity and lysophosphatidylcholine acyltransferase (LPCAT) activity.^20^ PRDX3 is involved in research in multiple fields, including cancer and brain diseases.^21^ PRDX3 is a multifunctional protein that participates in preventing oxidative damage, regulating cell proliferation and intracellular signaling, and contributing to the malignant progression, recurrence, and death of tumors. As an endogenous antioxidant enzyme, PRDX3 promotes their malignant progression. The differential expression of glycosyltransferases in tumor cells has been studied as a predictive biomarker and potential therapeutic target. This study aims to explore the influence of PRDX3 on ferroptosis and oxidative stress of osteoarthritis cartilage injury and the possible underlying mechanism.^19-21^

## Material and Methods

### Osteoarthritis Patients Experiment

Patients with OA and normal volunteers were obtained from the West China Hospital of Sichuan. Written informed consent was obtained from the patient who agreed to take part in the study. This study was approved by the Ethics Committee of the West China Hospital of Sichuan (No.2022-236; date:July 24, 2022). The basic information about this study was shown in Table 1.

### Real-time Polymerase Chain Reaction

Total RNAs were isolated with Trizol reagent (Beyotime) and cDNA was synthesized using PrimeScipt RT Master Mix (Takara). Quantitative polymerase chain reaction was performed with the ABI Prism 7500 sequence detection system according to the Prime-ScriptTM RT detection kit. Relative levels of the sample mRNA expression were calculated and expressed as 2 −ΔΔCt.

### In Vitro Model

SW982 cells were maintained in Dulbecco’s Modified Eagle Medium (DMEM) (Gibco) with 10% FBS (Gibco) under a humidified 5% (v/v) CO_2_ atmosphere at 37°C. SW982 cells were transfected with PRDX3 plasmid (sc-419122, Santa Cruz Biotechnology, Inc.) or si-PRDX3 plasmid (sc-40834, Santa Cruz Biotechnology, Inc.) using Lipofectamine 3000. After 48 hours, cells were treated with 200 ng/mL lipopolysaccharide (LPS) (Sigma, St. Louis, MO, USA) in a 10-cm Petri dish for 24 hours after transfection at 48 hours. The number of every group was 3.

### Animals

C57BL/6 mice (5-6 weeks, 19-21 g, male) were maintained in standard plastic rodent cages and maintained in a controlled environment, fed with food and water ad libitum. Adjuvant arthritis was induced on day 0 of the experiment by a single subcutaneous injection of 0.02 mL of complete Freund’s adjuvant per 1.0 mL sterile, non-metabolizable oils, into the plantar surface of the right hind paw of the mice. The number of every group was six.

### Histological Analysis

The tissue samples were fixed in 4% paraformaldehyde and executed histological analysis. Tissue samples were observed using a fluorescence microscope (Zeiss Axio Observer A1, Germany).

### Cell Viability Assay and Lactic dehydrogenase (LDH) Activity Assay

Cell viability was determined by the CCK-8 assay (C0037, Beyotime) or EdU kit (C0075S, Beyotime) or LDH activity (C0016, Beyotime) as literature.^22^ Absorbance was measured at 450 nm using a fluorescent reader (Synergy H1 Microplate Reader, Bio Tek, Winooski). Images were visualized using a fluorescence microscope (Olympus).

Malondialdehyde, SOD, GSH-PX, GSH, JC-1 disaggregation and calcien-AM/CoCl2 was determined by kits (S0131S, S0101S, S0053, S0057S, C2003S, C2009S, Beyotime).

### Western Blotting Analysis and Immunofluorescence

Western Blotting Analysis and Immunofluorescence were executed as literature.^23^ The membranes were PRDX3 (ab128953, 1:1000, Abcam), SIRT3 (ab217319, 1:1000, Abcam), GPX4 (ab262509, 1:1000, Abcam), and β-actin (GB15003-100, 1:1000, Servicebio) at 4°C overnight. The membranes were incubated with horseradish peroxidase-conjugated secondary antibodies (GB23303 or GB23301, 1:5000, Servicebio) for 1 h at 37°C. Protein was measured using an enhanced chemiluminescence system (ECL, Beyotime) and analyzed using an Image Lab 3.0 (BioRad Laboratories, Inc.).

Cells were incubated at 4°C overnight after blocking with PBS supplemented with 5% BSA for 2 hours at room temperature. The cells were incubated with Alexa Fluor® 488 /555 secondary antibody for 2 hours at room temperature and then stained with 4’,6-diamidino-2-phenylindole (DAPI) for 15 minutes in the dark.

### Coimmunoprecipitation Assay, The m6A Quantification, Luciferase Reporter Assay, and M6A-RNA Immunoprecipitation Assay

ChIP-qPCR, m6A quantification, Luciferase reporter, and MeRIP experiment were performed as previously described.^24^ The immunoprecipitated RNA was digested, purified, and further analyzed by qPCR. m6A mRNA levels are colourimetrically measured by enzyme-linked immunosorbent assay (ELISA) assay with an EpiQuik m6A RNA Methylation Quantification kit and Renilla luciferase activities were determined using a Dual-Luciferase Assay kit (Promega). For MeRIP, poly (A) + mRNA was isolated using the Dynabeads mRNA Direct Purification Kit (B518710, Sangon Biotech (Shanghai) Co., Ltd.).

### Statistical Analysis

*P* values lower than .05 were considered significant. The number of experimental repetitions = 3. Data were expressed as the mean ± SEM using GraphPad Prism 8 (GraphPad Software; San Diego, California, USA) and evaluated using one-way ANOVA followed by Tukey’s posttest.

## Results

### PRDX3 Expression Was Downregulated in the Osteoarthritis Model

**Figure 1.**
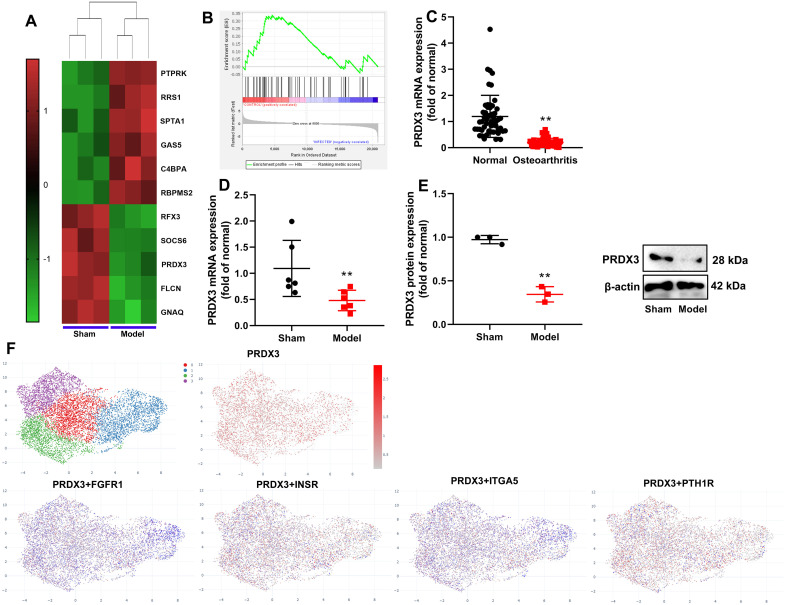
PRDX3 expression in a model of osteoarthritis was downregulated. Heat map (A), GO-BP enrichment analysis for PRDX3 (B), PRDX3 mRNA expression in patients with OA; PRDX3 mRNA and protein expression (D and E) in mice model with OA; Single-cell analysis revealed that the *PRDX3* gene was expressed in bone cells in patients with OA (F); **P < .01 vs normal or sham. OA, osteoarthritis.

This study investigated the expression levels of PRDX3 in the osteoarthritis model. Heat map and Gene Ontology - Biological Process (GO-BP) enrichment analysis demonstrated that PRDX3 mRNA expression was downregulated in osteoarthritis patients (Figure 1A-C). In the mice model of osteoarthritis, both PRDX3 mRNA and protein expression levels were suppressed (Figure 1D and E). Subsequently, single-cell analysis indicated that the *PRDX3* gene was expressed in bone cells in osteoarthritis patients (Figure 1F).

### Sh-PRDX3 Promoted Cartilage Injury in the Osteoarthritis Mouse Model Via Induction of Oxidative Stress

**Figure 2.**
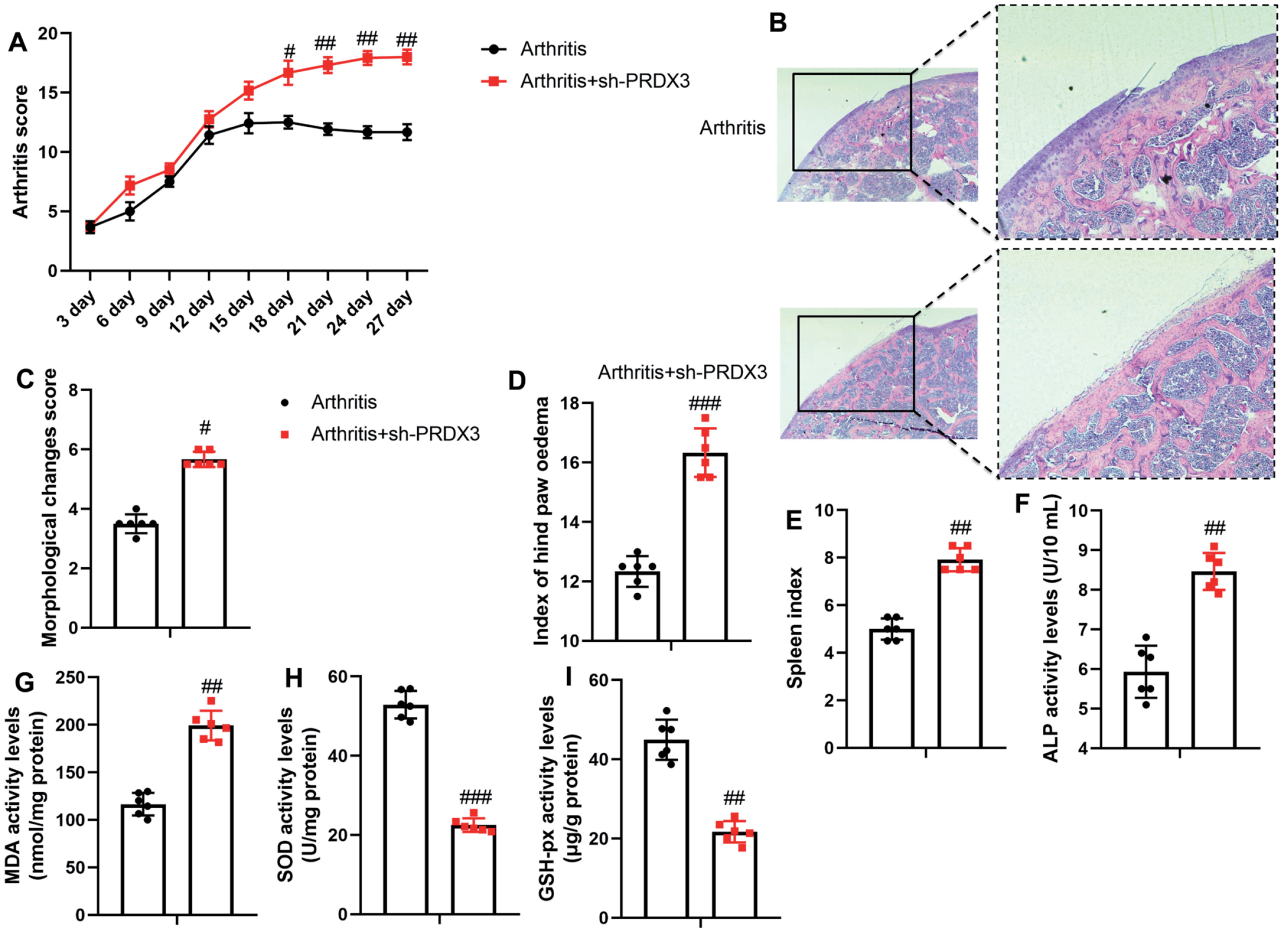
Sh-PRDX3 promoted osteoarthritis cartilage injury in mice models through the induction of oxidative stress. Arthritis score (A), cartilage injury (HE staining, B), morphopathological changes score (C), index of hind paw edema (D), spleen index (E), ALP and MDA activity levels (F, G), SOD and GSH-PX activity levels (H, I) in models of OA by sh-PRDX3. ^#^*P* < .05, ^##^*P* < .01, ^###^*P* < .001 vs arthritis.

Next, the sh-PRDX3 virus was utilized to reduce PRDX3 expression in an osteoarthritis mouse model. Sh-PRDX3 elevated the arthritis score and cartilage injury (hematoxylin-eosin (HE) staining), increased the morphological changes score, hind paw edema index, and spleen index in osteoarthritis mice (Figure 2A-E). Furthermore, the sh-PRDX3 virus increased the activity levels of Alkaline Phosphatase (ALP) and MDA while reducing the activity levels of SOD and GSH-PX in the articular tissue of osteoarthritis mice (Figure 2F-I).

### PRDX3 Amelioratedoxidative Stress in an In Vitro Osteoarthritis Model

**Figure 3.**
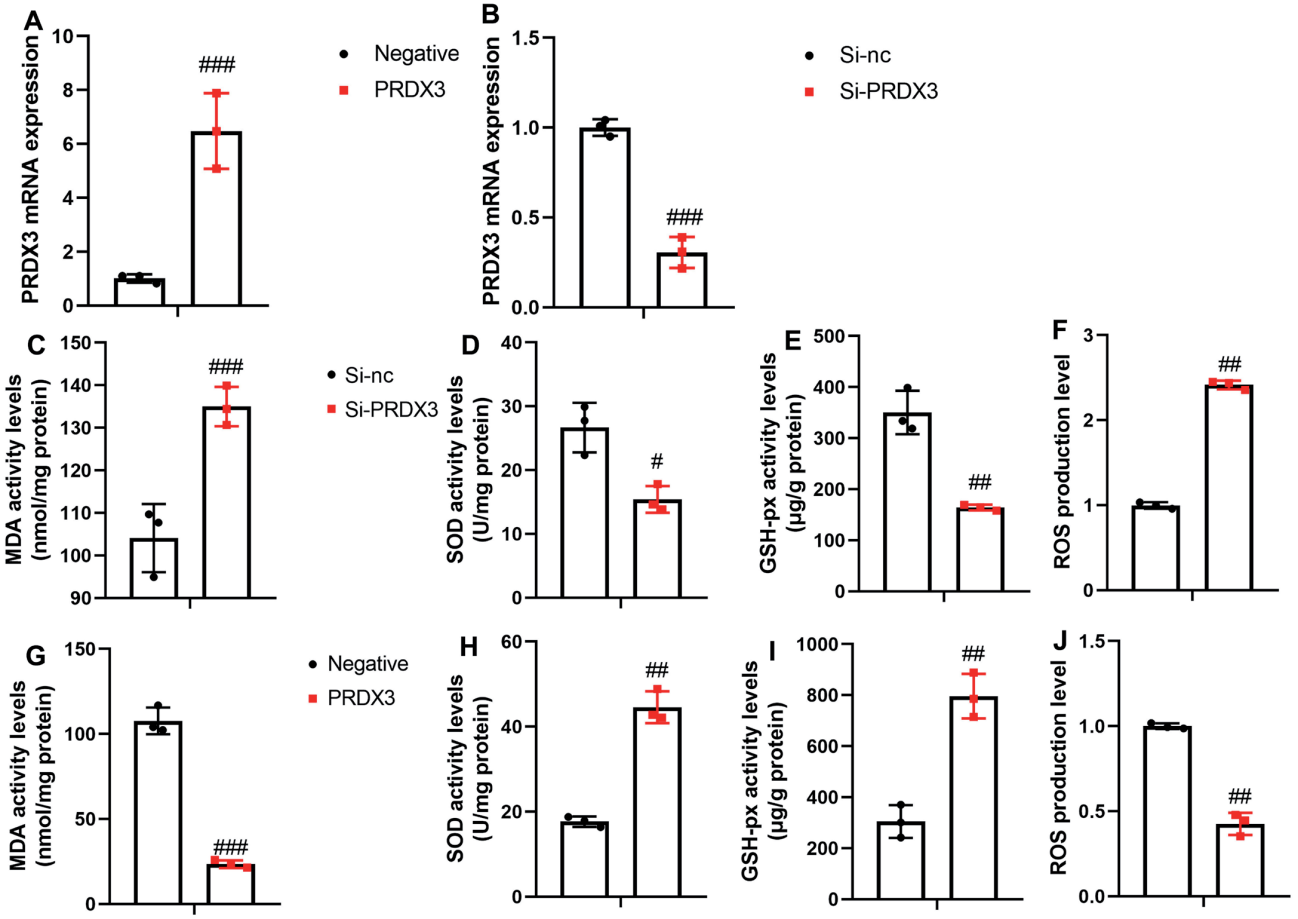
PRDX3 reduced oxidative stress in vitro model of osteoarthritis. PRDX3 mRNA expression (A, B) in an in vitro model of osteoarthritis; MDA, SOD, GSH-PX, ROS levels (C, D, E, F) in an in vitro model of osteoarthritis by si-PRDX3; MDA, SOD, GSH-PX, ROS levels (G, H, I, J) in the in vitro model of osteoarthritis by PRDX3. ^#^*P* < .05, ^##^*P* < .01, ^###^*P* < .001 vs si-nc or negative.

We transfected PRDX3 or si-PRDX3 plasmid to enhance or inhibit PRDX3 mRNA expression in the in vitro model, respectively (Figure 3A and B). Si-PRDX3 increased the MDA activity levels and ROS production levels while decreasing SOD and GSH-PX activity levels in the in vitro model (Figure 3C-F). PRDX3 upregulation reduced MDA activity levels and ROS production levels and increased SOD and GSH-PX activity levels in the in vitro model (Figure 3G-J).

### PRDX3 Alleviates Ferroptosis In Vitro Model or Osteoarthritis Model

**Figure 4.**
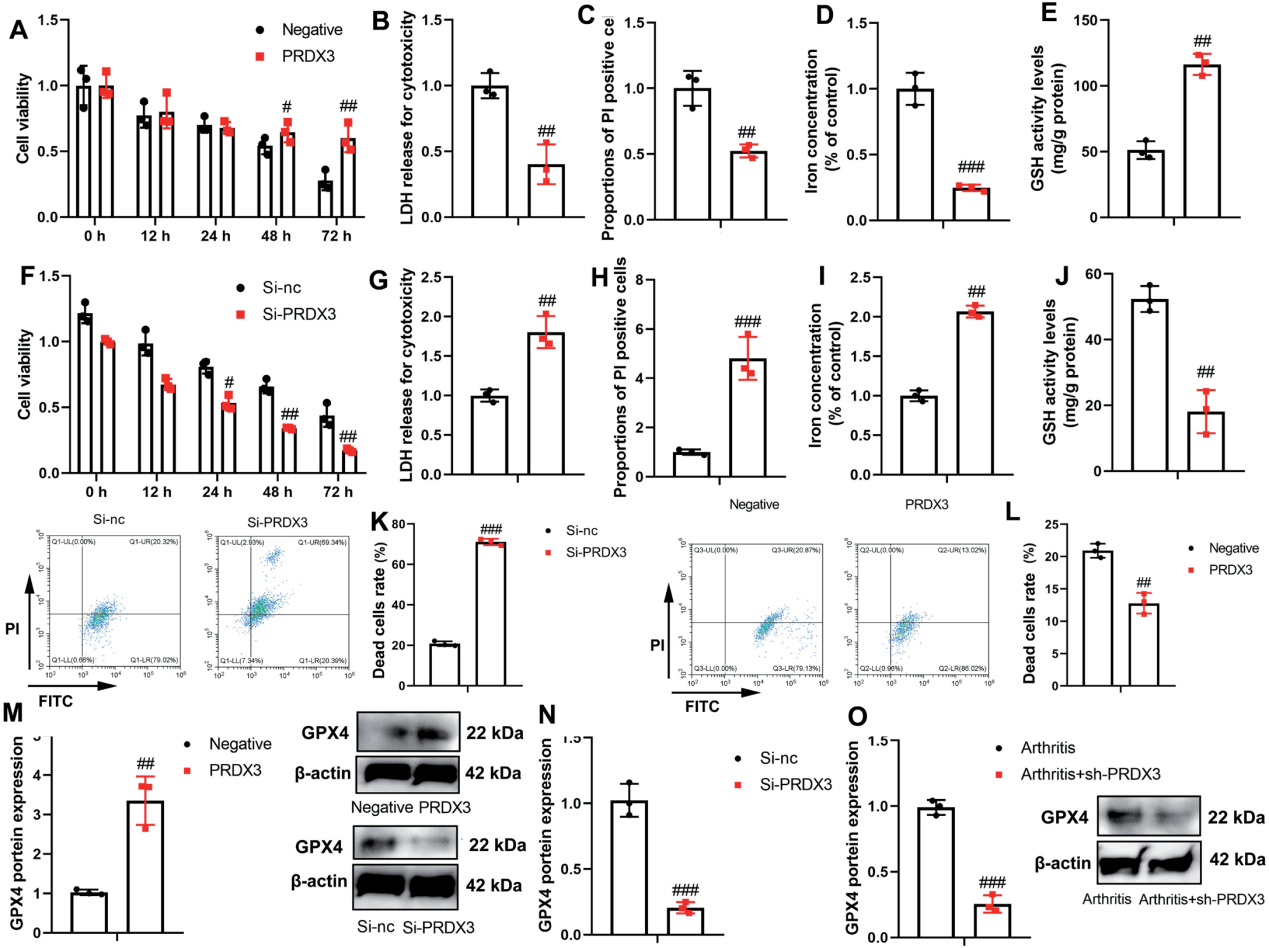
PRDX3 reduced ferroptosis in an in vitro model or mice model of osteoarthritis. Cell proliferation (A), LDH and PI levels (B, C), iron content (D), GSH activity level (E) in an in vitro model of osteoarthritis by PRDX3; Cell proliferation (F), LDH and PI levels (G, H), iron content (I), GSH activity level (J) in an in vitro model of osteoarthritis by Si-PRDX3; Dead cell rate in vitro model of osteoarthritis by Si-PRDX3 (K) or PRDX3 (L); GPX4 protein expression in an in vitro model of osteoarthritis by PRDX3 (M) or si-PRDX3 (N) or in a mice model of osteoarthritis by sh-PRDX3 (O). ^#^*P* < .05, ^##^*P* < .01, ^###^*P* < .001 vs si-nc or negative or arthritis.

Subsequently, PRDX3 up-regulation promoted cell growth and increased GSH activity levels, and decreased LDH activity, the proportion of Propidium Iodide (PI) positivity cells, and iron concentration in the in vitro osteoarthritis model (Figure 4A-E). This process was reversed by the si-PRDX3 plasmid (Figure 4F-J). Moreover, si-PRDX3 promoted the rate of dead cells rate, whereasPRDX3 upregulation reduced the rate of dead cells rate in the in vitro osteoarthritis model (Figure 4K and L). PRDX3 up-regulation induced GPX4 protein expression and si-PRDX3 suppressed GPX4 protein expression in the in vitro osteoarthritis model (Figure 4M and N). In the mice osteoarthritis model, PRDX3 up-regulation also suppressed GPX4 protein expression in the articular tissue of osteoarthritis mice (Figure 4O).

### PRDX3 Suppressed Reactive Oxygen Species Accumulation and Mitochondria-Dependent Ferroptosis In Vitro and In Vivo Models of Osteoarthritis

**Figure 5.**
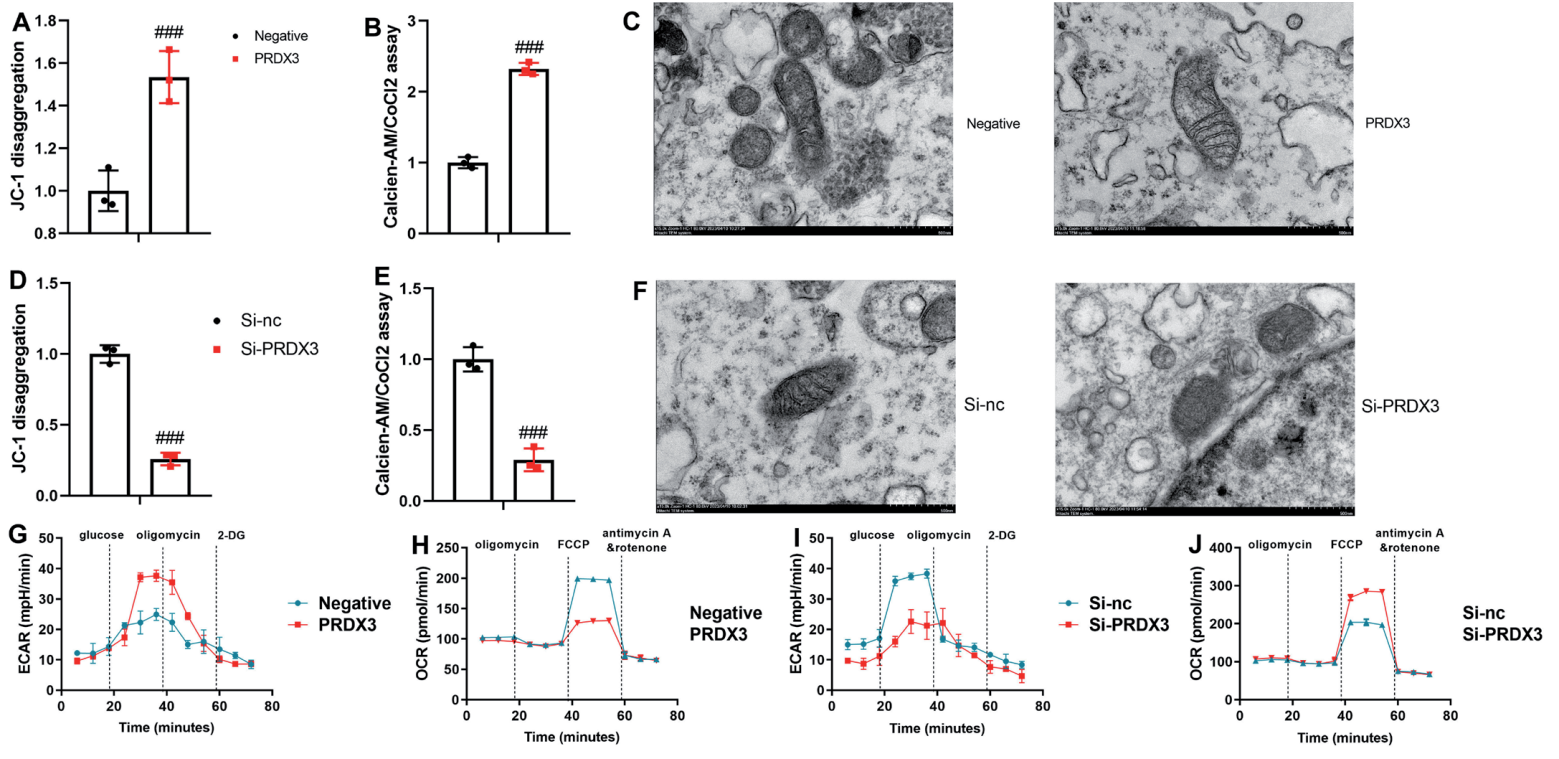
PRDX3 suppressed ROS accumulation and mitochondria-dependent ferroptosis in an in vitro model or mice model of osteoarthritis. JC-1 disaggregation (A), calcein-AM/CoCl2 (B), mitochondrial damage (C) in an in vitro model of osteoarthritis by PRDX3; JC-1 disaggregation (D), calcein-AM/CoCl2 (E), mitochondrial damage (F) in an in vitro model of osteoarthritis by si-PRDX3; ECAR level (G) and OCR levels (H) in an in vitro model of osteoarthritis by PRDX3; ECAR level (I) and OCR levels (J) in an in vitro model of osteoarthritis by si-PRDX3; ^###^*P* < .001 vs si-nc or negative.

In the in vitro osteoarthritis model, PRDX3 up-regulation enhanced JC-1 disaggregation and calcein-AM/CoCl2, and alleviated mitochondrial damage (Figure 5A-C). In contrast, si-PRDX3 decreased JC-1 disaggregation and calcein-AM/CoCl2, and promoted mitochondrial damage in the in vitro model of osteoarthritis (Figure 5D-F). Moreover, PRDX3 up-regulation increased Extracellular acidification rate (ECAR) levels, while decreasing oxygen consumption rates (OCR) levels in the in vitro osteoarthritis model (Figure 5G and H). Conversely, si-PRDX3 reduced ECAR levels and increased OCR levels in the in vitro osteoarthritis model (Figure 5I-J).

### PRDX3 Induced the Expression Level of Sirtuin-3 (SIRT3) in the Osteoarthritis Model

**Figure 6.**
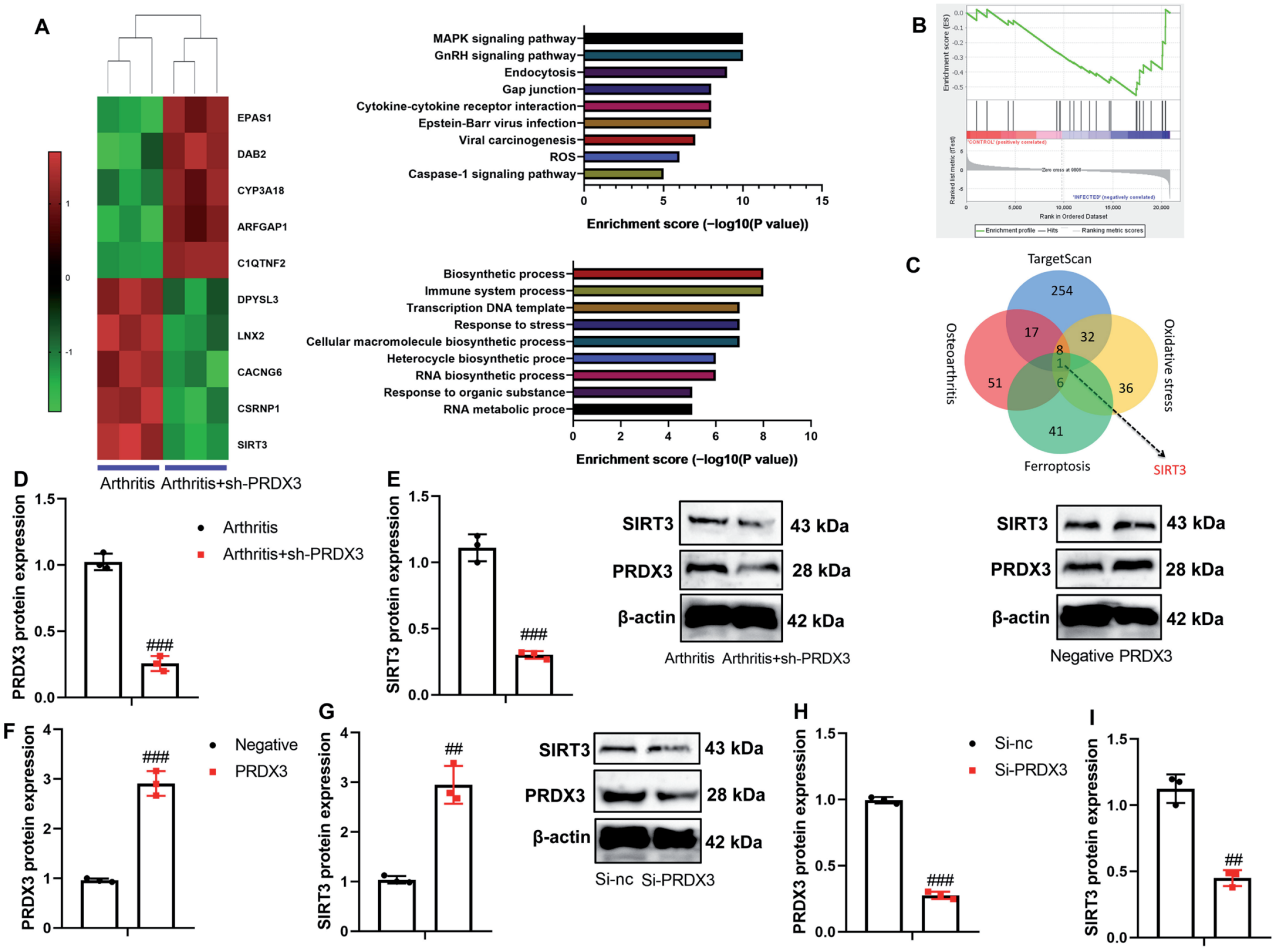
PRDX3 induced SIRT3 expression level in a model of osteoarthritis. Heat map (A), GO-BP enrichment analysis for SIRT3 (B), result analysis for SIRT3 (C), PRDX3/SIRT3 protein expression (D, E) in mice model of osteoarthritis by sh-PRDX3; PRDX3/SIRT3 protein expression (F, G) in vitro model of osteoarthritis by PRDX3; PRDX3/SIRT3 protein expression (H, I) in vitro model of osteoarthritis by si-PRDX3; ^##^*P* < .01, ^###^*P* < .001 vs si-nc or negative or arthritis.

Subsequently, the mechanism of PRDX3 was investigated in mitochondria-dependent ferroptosis in osteoarthritis using a gene chip. Sh-PRDX3 down-regulated SIRT3 expression in the articular tissue of osteoarthritis mice (Figure 6A-C). Moreover, sh-PRDX3 suppressed the protein expressions of both PRDX3 and SIRT3 in the articular tissue of osteoarthritis mice (Figure 6D and E). In the in vitro osteoarthritis model, PRDX3 up-regulation led to an increase in the protein expressions of PRDX3 and SIRT3, while si-PRDX3 suppressed PRDX3 and SIRT3 protein expressions (Figure 6F-I).

**Figure 7.**
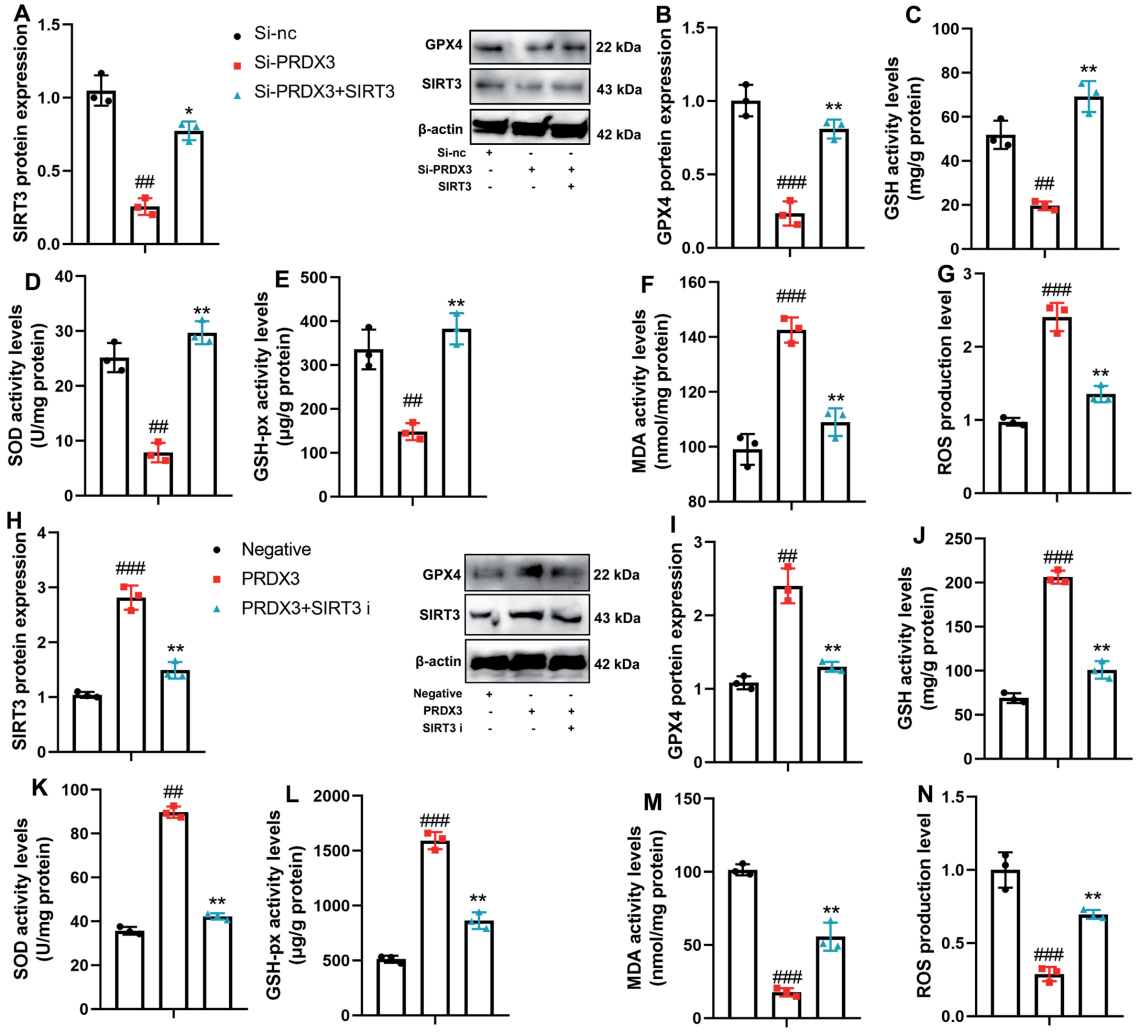
SIRT3 regulated the effects of PRDX3 on oxidative stress in vitro model of osteoarthritis. SIRT3/GPX4 protein expression (A, B), GSH/SOD/GSH-PX/MDA/ROS levels (C, D, E, F, G) in an in vitro model of osteoarthritis by si-PRDX3+ SIRT3; SIRT3/GPX4 protein expression (H, I), GSH/SOD/GSH-PX/MDA/ROS levels (J, K, L, M, N) in an in vitro model of osteoarthritis by PRDX3+ SIRT3 inhibitor; ^##^*P* < .01, ^###^*P* < .001 vs si-nc or negative; ^*^P < .05, ^**^P < .01 vs si-PRDX3 or PRDX3.

In this study, the SIRT3 agonist (0.05 μM of SRT 1720 dihydrochloride) was used to induce SIRT3 and GPX4 protein expressions and attenuate the effects of si-PRDX3 on ROS accumulation in the in vitro osteoarthritis model (Figure 7A-G). Meanwhile, the SIRT3 inhibitor (10 μM of SRT AGK2) suppressed SIRT3 and GPX4 protein expressions and inhibited the effects of PRDX3 on ROS accumulation in the in vitro osteoarthritis model (Figure 7H-N).

**Figure 8.**
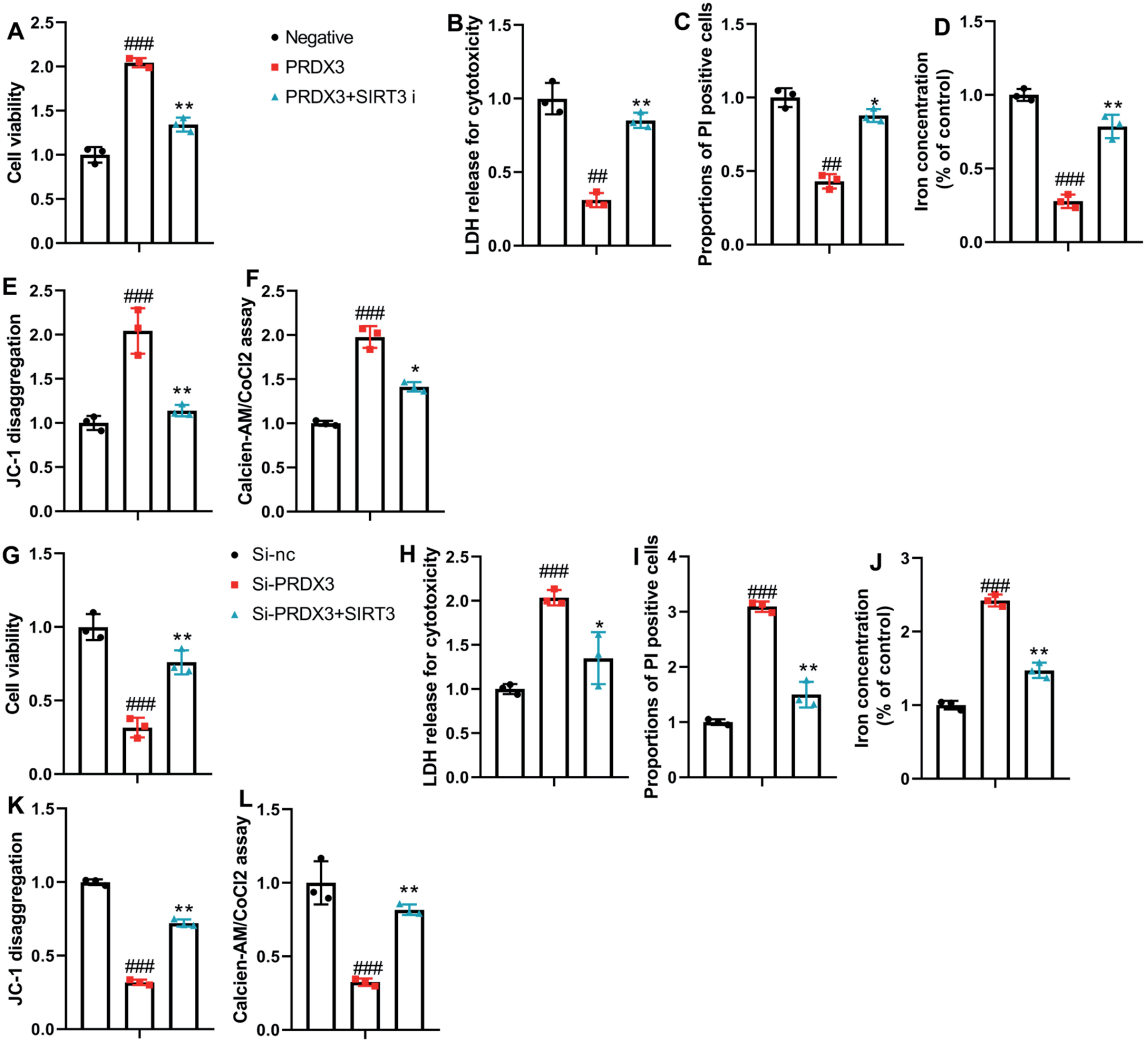
SIRT3 regulated the effects of PRDX3 on ferroptosis in an in vitro model of osteoarthritis. Cell proliferation (A), LDH and PI levels (B, C), iron content (D), JC-1 disaggregation (E), calcein-AM/CoCl2 (F) in the in vitro model of osteoarthritis by PRDX3+ SIRT3 inhibitor; Cell proliferation (G), LDH and PI levels (H, I), iron content (J), JC-1 disaggregation (K), calcein-AM/CoCl2 (L) in the in vitro model of osteoarthritis by si-PRDX3 + SIRT3; ^##^*P* < .01, ^###^*P* < .001 vs si-nc or negative; *P < .05, **P < .01 vs si-PRDX3 or PRDX3.

Next, the SIRT3 inhibitor also promoted ferroptosis and mitochondrial damage in the in vitro osteoarthritis model with the presence of PRDX3 (Figure 8A-F). However, the SIRT3 agonist reduced ferroptosis and mitochondrial damage in the in vitro osteoarthritis model with the intervention of si-PRDX3 (Figure 8G-L).

### PRDX3 Reduced SIRT3 Ubiquitination in the Model of Osteoarthritis

**Figure 9.**
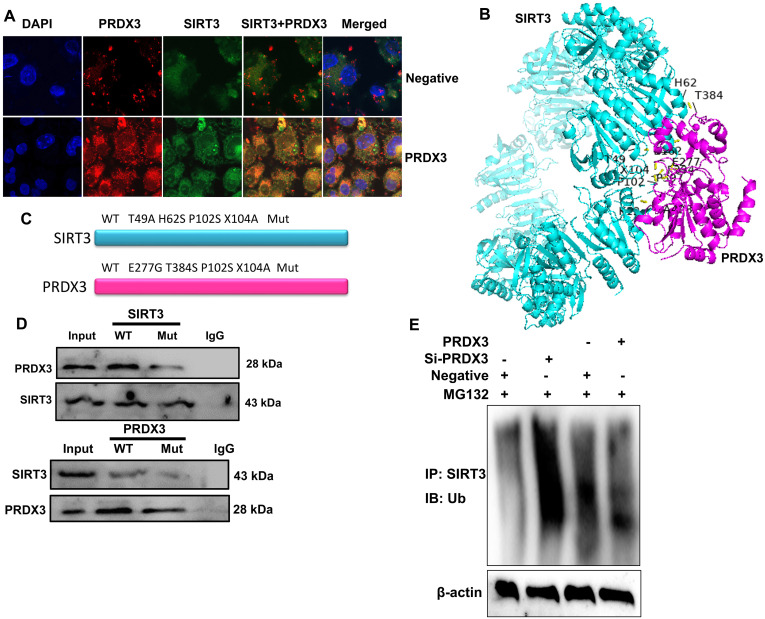
PRDX3 reduced SIRT3 ubiquitination in a model of osteoarthritis. PRDX3/SIRT3 expression (immunofluorescence, A); 3D structure for PRDX3 protein interlinking with SIRT3 protein (B); WT/Mutation site of PRDX3 or SIRT3 (C); IP assay for PRDX3 protein interlinking with SIRT3 protein (C); SIRT3 ubiquitination (D).

Subsequently, the mechanism of PRDX3 on SIRT3 in osteoarthritis was explored. Immunofluorescence results indicated that PRDX3 up-regulation induced the expression of both PRDX3 and SIRT3 in the in vitro model of osteoarthritis (Figure 9A). Through the application of 3D model prediction, it was discovered that the PRDX3 protein interacted with the SIRT3 protein (Figure 9B). Immunoprecipitation (IP) analysis demonstrated that SATB2 WT protein interacted with the SIRT3 WT protein; however, the WT PRDX3 protein did not interact with the Wnt SIRT3 protein, and the Mut PRDX3 Mut protein did not link with the WT SIRT3 protein (Figure 9C-D). PRDX3 up-regulation reduced SIRT3 ubiquitination, and si- PRDX3 induced SIRT3 ubiquitination in the in vitro model of osteoarthritis (Figure 9E).

### In the Model of Cartilage Injury in Osteoarthritis, METTL3-Mediated m6A Modification Decreases the mRNA Stability of PRDX3

**Figure 10.**
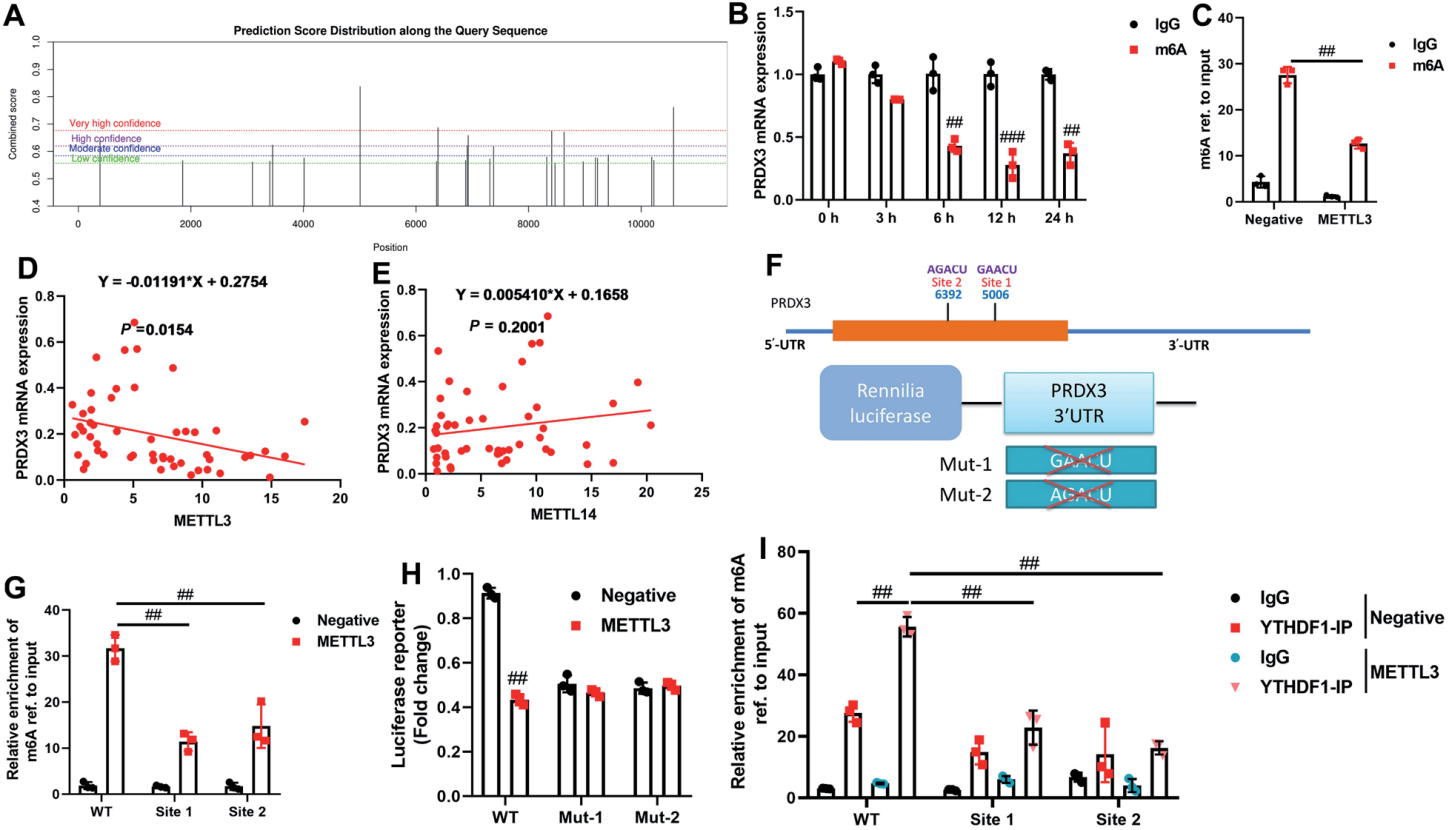
METTL3-mediated m6A modification decreases PRDX3 mRNA stability in a model of osteoarthritis cartilage injury. Methylation modification sites near (A), PRDX3 mRNA expression (B), the stability of PRDX3 mRNA (C), PRDX3/METTL3 levels in patients with osteoarthritis (D), PRDX3/METTL14 levels in patients with osteoarthritis (E), 3′-untranslated region (UTR) of PRDX3 (F), m6A modification (G), luciferase activity level (H), modification (F), m6A modification by YTHDF1 (I). ^##^*P* < .01, ^###^*P* < .001.

Subsequently, the molecular mechanism by which METTL3 mediates ferroptosis in osteoarthritis through PRDX3 was investigated. PRDX3 possesses multiple suspected methylation modification sites in proximity (Figure 10A). Interestingly, the m6A-specific antibody significantly inhibited PRDX3 mRNA expression levels (Figure 10B). In osteoarthritis, METTL3 remarkably decreased the stability of PRDX3 mRNA (Figure 10C).

In osteoarthritis patients, the mRNA expression of PRDX3 was negatively correlated with METTL3 mRNA expression, but not with METTL14 mRNA expression (Figure 10D-E). There are m6A sites in the 3′-untranslated region (UTR) of PRDX3 (Figure 10F). METTL3 was significantly enriched in PRDX3 m6A modification at sites 1 and 2 (Figure 10G). METTL3 reduced the luciferase activity level by wild-type (WT) of PRDX3, while the mutant (Mut) PRDX3 did not (Figure 10H). The knockdown of YTHDF1 considerably alleviated the METTL3-induced reduction of PRDX3 m6A modification, suggesting that YTHDF1 knockdown could regulate PRDX3 m6A modification (Figure 10I). These findings revealed that METTL3-mediated m6A modification decreases PRDX3 stability in osteoarthritis via YTHDF1.

## Discussion

Patients with OA experience joint pain and functional impairment, which in turn reduces their quality of life. The increasing prevalence of OA is due to factors such as an aging population, depression, excessive alcohol consumption, trauma, and elevated joint load, and this prevalence is expected to keep rising in the coming decades.^25^ Other studies have found that abnormal conditions like bone marrow lesions, meniscus, and cartilage injuries raise the incidence of OA more significantly than common risk factors.^26^ In the early stages of the disease, symptoms such as joint swelling and pain may occur; in the later stages, functional limitations may develop, increasing the risk of OA-related disability.^27^ The knee joint is one of the joints most frequently affected by osteoarthritis, and approximately 30% of people over 45 years old have knee osteoarthritis (KOA).^28^ Traditional treatment methods for KOA mainly consist of surgical treatments, such as autologous chondrocyte implantation, and non-surgical treatments, such as platelet-rich plasma or hyaluronic acid injection.^29^ However, these treatment methods can only alleviate the symptoms of patients and cannot repair the joint cartilage damage caused by OA.^30^ Therefore, it is still necessary to develop new treatment strategies to improve joint cartilage injuries. In this study, PRDX3 mRNA expression was down-regulated in the OA model. Sh-PRDX3 promoted cartilage injury in the mouse model of OA through the induction of oxidative stress. Li et al^31^ revealed that PRDX3 ameliorates osteoarthritis. Furthermore, PRDX3 is involved in the entire process of cartilage injury in osteoarthritis.

It is associated with cellular glycolysis in bone tissue. The occurrence of OA cartilage injury is also related to the abnormal ferroptosis of chondrocytes.^32^ Intra-articular injection of ferroptosis inhibitors to inhibit the ferroptosis of chondrocytes can alleviate cartilage injury and delay the progression of OA.^33^ Studies have shown that inhibiting ferroptosis in chondrocytes is an effective approach to delaying cartilage degeneration in osteoarthritis. Ferroptosis, which is iron-dependent cell death, is a type of programmed cell death caused by excessive lipid peroxidation accumulation resulting in membrane rupture.^34^ GPX4 is a glutathione-regulated lipid repair enzyme that inhibits ferroptosis by reducing ROS.^24^ Currently, PRDX3 has been shown to suppress ROS accumulation and mitochondria-dependent ferroptosis in both the in vitro model and the mouse model of osteoarthritis. Xu et al^35^ showed that PRDX3 mediated oxidative stress in ovarian cancer stem cells. Cui et al^21^ reported that PRDX3 served as a ferroptosis marker in chronic liver diseases.^21^ These data suggested that PRDX3 can reduce ferroptosis and oxidative stress in the osteoarthritis model by a mitochondria-dependent mechanism. In this study, only PRDX3’s ability to reduce ferroptosis and oxidative stress of SW982 cells in osteoarthritis was analyzed. However, it is more relevant to study the cartilage in OA. This is one limitation of this study. The function of PRDX3 on the cartilage in OA will be researched.

The SIRT3/SOD2 signaling pathway is a mitochondrial-associated antioxidant stress pathway. Activation of this pathway can suppress ROS production, ameliorate oxidative stress and mitochondrial function, and relieve the damage to airway epithelial cells induced by cigarette extract (CES). Meanwhile, it was discovered that in the osteoarthritis model, PRDX3 induced SIRT3 expression level by inhibiting the ubiquitination of SIRT3. Tan et al^36^ showed that the Sirt3/Prdx3 pathway alleviates mitochondrial dysfunction caused by hypoxia/reoxygenation in cardiomyocytes.^36^ However, this experiment demonstrated that in the osteoarthritis model, PRDX3 induced SIRT3 expression to control ROS accumulation and mitochondria-dependent ferroptosis. In this study, only one ferroptosis regulator, GPX4, was analyzed which was insufficient. The relationship between PRDX3 and other ferroptosis regulators will be analyzed.

This article provides an overview of m6A from 3 aspects: N6 methyladenine (m6A) methyltransferase, m6A demethylase, and m6A reading protein.^37^ It also summarizes the role of m6A RNA methylation modification in the pathogenesis of rheumatoid arthritis, osteoarthritis, and systemic lupus erythematosus.^38,39^ In the model of osteoarthritis cartilage injury, METTL3-mediated m6A modification reduces PRDX3 mRNA stability.

**Figure 11.**
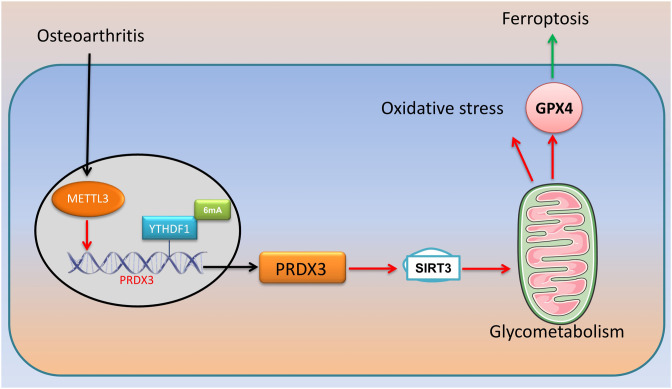
Methylation of PRDX3 expression alleviate ferroptosis and oxidative stress in patients with osteoarthritis cartilage injury.

In conclusion, in the model of osteoarthritis cartilage injury, METTL3-mediated m6A modification decreases PRDX3 mRNA stability. In the osteoarthritis model, PRDX3 induced SIRT3 expression to regulate ROS accumulation and mitochondria-dependent ferroptosis (Figure 11). Further elucidation of the role of PRDX3 and its relationship with the regulation of the SIRT3 pathway can facilitate the understanding of ROS accumulation and mitochondria-dependent ferroptosis and improve the clinical management of osteoarthritis. Targeting METTL3 or PRDX3 is thus a potentially effective therapeutic strategy for patients with osteoarthritis cartilage injury.

## Figures and Tables

**Table 1.** Basic Information of This Study

Study	Normal Volunteer	Patients with OA
Number	52	52
Age	54.85 ± 14.28	56.67 ± 15.09
Sex	Male: 33; Female: 19	Male: 31; Female: 21
Type of OA		
Unilateral		36
Bilateral		16
Disease duration (years)		5.02 ± 1.97

OA, osteoarthritis.

## Data Availability

The data that support the findings of this study are available on request from the corresponding author.

## References

[1] Gupta P Jamra A Prakash S Gupta S Bharti A . Evaluating the efficacy of platelet-rich plasma in treating primary knee osteoarthritis: a prospective interventional study. Cureus. 2024;16(10):e71415. (10.7759/cureus.71415) 39539882 PMC11558281

[2] Bijlsma JWJ Berenbaum F Lafeber FPJG . Osteoarthritis: an update with relevance for clinical practice. Lancet. 2011;377(9783):2115 2126. (10.1016/S0140-6736(11)60243-2) 21684382

[3] Huang D Wang H Wang S Yu T Zhou L . Associations between urinary phytoestrogen mixed metabolites and osteoarthritis risk. PLoS One. 2024;19(11):e0313675. (10.1371/journal.pone.0313675) 39541342 PMC11563356

[4] Liao J Gu Q Liu Z , et al. Edge advances in nanodrug therapies for osteoarthritis treatment. Front Pharmacol. 2024;15:1402825. (10.3389/fphar.2024.1402825) 39539625 PMC11559267

[5] King LK Stanaitis I Hung V , et al. National Institute of Health and care excellence clinical criteria for the diagnosis of knee osteoarthritis: A prospective diagnostic accuracy study in individuals with type 2 diabetes. Arthritis Care Res (Hoboken). 2025;77(5):623 630. (10.1002/acr.25464) 39542837 PMC12038217

[6] Liang H Si W Li L Yang K . Association between body roundness index and osteoarthritis: a cross-sectional analysis of NHANES 2011-2018. Front Nutr. 2024;11:1501722. (10.3389/fnut.2024.1501722) 39545042 PMC11560466

[7] Garofoli R Renard D Bessiene L Lefèvre-Colau MM . Shoulder osteoarthritis facilitating the diagnosis of acromegaly. BMJ Case Rep. 2024;17(11):e258545. (10.1136/bcr-2023-258545) 39542498

[8] Felson DT Lawrence RC Dieppe PA , et al. Osteoarthritis: new insights. Part 1: the disease and its risk factors. Ann Intern Med. 2000;133(8):635 646. (10.7326/0003-4819-133-8-200010170-00016) 11033593

[9] Hartnett DA Milner JD DeFroda SF . Osteoarthritis in the upper extremity. Am J Med. 2023;136(5):415 421. (10.1016/j.amjmed.2023.01.025) 36740213

[10] Macías-Hernández SI Morones-Alba JD Miranda-Duarte A , et al. Glenohumeral osteoarthritis: overview, therapy, and rehabilitation. Disabil Rehabil. 2017;39(16):1674 1682. (10.1080/09638288.2016.1207206) 27416338

[11] Taruc-Uy RL Lynch SA . Diagnosis and treatment of osteoarthritis. Prim Care. 2013;40(4):821 836, vii. (10.1016/j.pop.2013.08.003) 24209720

[12] Abramoff B Caldera FE . Osteoarthritis: pathology, diagnosis, and treatment options. Med Clin North Am. 2020;104(2):293 311. (10.1016/j.mcna.2019.10.007) 32035570

[13] Guo Z Lin J Sun K , et al. Deferoxamine alleviates osteoarthritis by inhibiting chondrocyte ferroptosis and activating the Nrf2 pathway. Front Pharmacol. 2022;13:791376. (10.3389/fphar.2022.791376) 35359876 PMC8964096

[14] Miao Y Chen Y Xue F , et al. Contribution of ferroptosis and GPX4’s dual functions to osteoarthritis progression. EBiomedicine. 2022;76:103847. (10.1016/j.ebiom.2022.103847) 35101656 PMC8822178

[15] Wan Y Shen K Yu H Fan W . Baicalein limits osteoarthritis development by inhibiting chondrocyte ferroptosis. Free Radic Biol Med. 2023;196:108 120. (10.1016/j.freeradbiomed.2023.01.006) 36657732

[16] Guan Z Jin X Guan Z Liu S Tao K Luo L . The gut microbiota metabolite capsiate regulate SLC2A1 expression by targeting HIF-1α to inhibit knee osteoarthritis-induced ferroptosis. Aging Cell. 2023;22(6):e13807. (10.1111/acel.13807) 36890785 PMC10265160

[17] Pu Z Sui B Wang X Wang W Li L Xie H . The effects and mechanisms of the anti-COVID-19 traditional Chinese medicine, Dehydroandrographolide from Andrographis paniculata (Burm.f.) Wall, on acute lung injury by the inhibition of NLRP3-mediated pyroptosis. Phytomedicine. 2023;114:154753. (10.1016/j.phymed.2023.154753) 37084628 PMC10060206

[18] Al-Hetty HRAK Abdulameer SJ Alghazali MW Sheri FS Saleh MM Jalil AT . The role of ferroptosis in the pathogenesis of osteoarthritis. J Membr Biol. 2023;256(3):223 228. (10.1007/s00232-023-00282-0) 36920529

[19] Sun R Tian X Li Y , et al. The m6A reader YTHDF3-mediated PRDX3 translation alleviates liver fibrosis. Redox Biol. 2022;54:102378. (10.1016/j.redox.2022.102378) 35779442 PMC9287738

[20] Ramasamy P Larkin AM Linge A , et al. PRDX3 is associated with metastasis and poor survival in uveal melanoma. J Clin Pathol. 2020;73(7):408 412. (10.1136/jclinpath-2019-206173) 31771972

[21] Cui S Ghai A Deng Y , et al. Identification of hyperoxidized PRDX3 as a ferroptosis marker reveals ferroptotic damage in chronic liver diseases. Mol Cell. 2023;83(21):3931 3939.e5. (10.1016/j.molcel.2023.09.025) 37863053 PMC10841858

[22] Xiao H Wang J Yuan L Xiao C Wang Y Liu X . Chicoric acid induces apoptosis in 3T3-L1 preadipocytes through ROS-mediated PI3K/Akt and MAPK signaling pathways. J Agric Food Chem. 2013;61(7):1509 1520. (10.1021/jf3050268) 23363008

[23] Pu Z Shen C Zhang W Xie H Wang W . Avenanthramide C from oats protects pyroptosis through dependent ROS-induced mitochondrial damage by PI3K ubiquitination and phosphorylation in pediatric pneumonia. J Agric Food Chem. 2022;70(7):2339 2353. (10.1021/acs.jafc.1c06223) 35119859

[24] Zhang W Liu Y Zhou J Qiu T Xie H Pu Z . Chicoric acid advanced PAQR3 ubiquitination to ameliorate ferroptosis in diabetes nephropathy through the relieving of the interaction between PAQR3 and P110α pathway. Clin Exp Hypertens. 2024;46(1):2326021. (10.1080/10641963.2024.2326021) 38525833

[25] Mao D Li S Li X , et al. Causal relationships between circulating immune cell traits and the risk of rheumatoid arthritis and osteoarthritis: A bidirectional Two-Sample Mendelian randomization study. Iran J Public Health. 2024;53(10):2307 2317. (10.18502/ijph.v53i10.16718) 39544856 PMC11557758

[26] McNally KR Summers S Stanton TR , et al. Exploring whether home-based neuromodulation can boost the analgesic effects of exercise in people with knee osteoarthritis: protocol for a double-blinded, pilot randomised controlled trial. BMJ Open. 2024;14(11):e090523. (10.1136/bmjopen-2024-090523) PMC11575249 39542463

[27] Wang J Peng L Yang M , et al. Is there a genetic relationship between blood glucose and osteoarthritis? A Mendelian randomization study. Diabetol Metab Syndr. 2024;16(1):274. (10.1186/s13098-024-01517-3) 39543708 PMC11562302

[28] Peoples BM Harrison KD Renfrow G , et al. Osteoarthritis and neurological disorder diagnoses in adults: a meta-analysis examining associations with Parkinson’s disease, multiple sclerosis, and Alzheimer’s disease. Cureus. 2024;16(10):e71458. (10.7759/cureus.71458) 39544560 PMC11560400

[29] Salamah AAS Láinez Ramos-Bossini AJ Khan KS Ruiz Santiago F . Diagnostic accuracy of magnetic resonance imaging (MRI) for symptomatic knee osteoarthritis: a scoping review. Quant Imaging Med Surg. 2024;14(11):8001 8011. (10.21037/qims-24-1544) 39544469 PMC11558480

[30] Liao X Chen X Zhou Y Xing L Shi Y Huang G . Added sugars and risk of osteoarthritis in adults: a case-control study based on National Health and Nutrition Examination survey 2007-2018. PLoS One. 2024;19(11):e0313754. (10.1371/journal.pone.0313754) 39541365 PMC11563403

[31] Li H Cao Y Chang C , et al. Knockdown of circSOD2 ameliorates osteoarthritis progression via the miR-224-5p/PRDX3 axis. J Orthop Surg Res. 2023;18(1):432. (10.1186/s13018-023-03880-9) 37312219 PMC10265860

[32] Xia L Gong N . Identification and verification of ferroptosis-related genes in the synovial tissue of osteoarthritis using bioinformatics analysis. Front Mol Biosci. 2022;9:992044. (10.3389/fmolb.2022.992044) 36106017 PMC9465169

[33] Yang J Hu S Bian Y , et al. Targeting cell death: pyroptosis, ferroptosis, apoptosis and necroptosis in osteoarthritis. Front Cell Dev Biol. 2021;9:789948. (10.3389/fcell.2021.789948) 35118075 PMC8804296

[34] Wang S Li W Zhang P , et al. Mechanical overloading induces GPX4-regulated chondrocyte ferroptosis in osteoarthritis via Piezo1 channel facilitated calcium influx. J Adv Res. 2022;41:63 75. (10.1016/j.jare.2022.01.004) 36328754 PMC9637484

[35] Xu S Liu Y Yang S , et al. FXN targeting induces cell death in ovarian cancer stem-like cells through PRDX3-Mediated oxidative stress. iScience. 2024;27(8):110506. (10.1016/j.isci.2024.110506) 39184439 PMC11342215

[36] Tan Q Dong W Wang Q Gao L . Dexmedetomidine alleviates Hypoxia/reoxygenation-induced mitochondrial dysfunction in cardiomyocytes via activation of Sirt3/Prdx3 pathway. Daru. 2024;32(1):189 196. (10.1007/s40199-024-00504-3) 38407745 PMC11087443

[37] Ye G Li J Yu W , et al. ALKBH5 facilitates CYP1B1 mRNA degradation via m6A demethylation to alleviate MSC senescence and osteoarthritis progression. Exp Mol Med. 2023;55(8):1743 1756. (10.1038/s12276-023-01059-0) 37524872 PMC10474288

[38] Lu Y Zhang H Pan H , et al. Expression pattern analysis of m6A regulators reveals IGF2BP3 as a key modulator in osteoarthritis synovial macrophages. J Transl Med. 2023;21(1):339. (10.1186/s12967-023-04173-9) 37217897 PMC10204300

[39] Zhai G Xiao L Jiang C , et al. Regulatory role of N6-methyladenosine (m6A) modification in osteoarthritis. Front Cell Dev Biol. 2022;10:946219. (10.3389/fcell.2022.946219) 35846376 PMC9282717

